# A Neural Network Model Can Explain Ventriloquism Aftereffect and Its Generalization across Sound Frequencies

**DOI:** 10.1155/2013/475427

**Published:** 2013-10-21

**Authors:** Elisa Magosso, Filippo Cona, Mauro Ursino

**Affiliations:** Department of Electrical, Electronic, and Information Engineering “Guglielmo Marconi”, University of Bologna, Via Venezia 52, 47521 Cesena, Italy

## Abstract

Exposure to synchronous but spatially disparate auditory and visual stimuli produces a perceptual shift of sound location towards the visual stimulus (ventriloquism effect). After adaptation to a ventriloquism situation, enduring sound shift is observed in the absence of the visual stimulus (ventriloquism aftereffect). Experimental studies report opposing results as to aftereffect generalization across sound frequencies varying from aftereffect being confined to the frequency used during adaptation to aftereffect generalizing across some octaves. Here, we present an extension of a model of visual-auditory interaction we previously developed. The new model is able to simulate the ventriloquism effect and, via Hebbian learning rules, the ventriloquism aftereffect and can be used to investigate aftereffect generalization across frequencies. The model includes auditory neurons coding both for the spatial and spectral features of the auditory stimuli and mimicking properties of biological auditory neurons. The model suggests that different extent of aftereffect generalization across frequencies can be obtained by changing the intensity of the auditory stimulus that induces different amounts of activation in the auditory layer. The model provides a coherent theoretical framework to explain the apparently contradictory results found in the literature. Model mechanisms and hypotheses are discussed in relation to neurophysiological and psychophysical data.

## 1. Introduction

Real world events generally impact most of our senses simultaneously. It is widely recognized that perception and behavior are driven by the integration of information from different sensory modalities [1]. Multisensory integration can improve detectability of external events, speed up responses, and solve ambiguous situations [2, 3]. How multisensory perception is accomplished at a neural level is a central research topic.

A substantial part of research has focused on visual-auditory interactions. Important information on audio-visual integration is provided by illusory phenomena. A well-known phenomenon occurs in case of visual-auditory spatial conflict: when a visual stimulus and an auditory stimulus are presented simultaneously but in disparate spatial positions, the perceived location of the sound is shifted towards the visual stimulus (i.e., visual bias of sound localization) [4–6]. This phenomenon is named *ventriloquism*, as it can explain the impression of a speaking puppet created by the illusionist.

Visual bias of sound location occurs not only with meaningful stimuli, but also with neutral and rudimental stimuli, such as flashes and beeps [5, 7, 8]. Translocation of the sound in these conditions is usually a percentage of the visual-auditory spatial disparity and is indicative of an automatic attraction of the sound toward the visual stimulus. The neural substrates for this phenomenon may involve a direct visual influence on the auditory cortex [9], mediated by the primary visual area [10, 11].

Exposure to a ventriloquism situation (adaptation phase) induces a recalibration of the auditory space: the perceived location of a unimodal auditory stimulus is shifted in the direction of the previously experienced visual stimulus [12]. This effect is named *ventriloquism aftereffect*. According to recent studies [13, 14], aftereffect may be acquired very quickly (even on milliseconds time scale), reflecting a phenomenon of rapid plasticity, and it can last until new spatial information between visual and auditory inputs becomes available [14].

Several studies have investigated the ventriloquism aftereffect [12, 15–19], with the main aim to assess whether the aftereffect is specific to the characteristics of the auditory stimuli used in the adaptation phase or instead it generalizes to a range of neighboring stimuli. Most of these studies were focused on the spectral characteristics of the auditory stimulus, and they investigated the extent of aftereffect generalization along the frequency dimension. To this purpose, during the adaptation phase, a pure tone stimulus at a specific frequency was applied (together with a spatially disparate visual stimulus); then, the magnitude of the aftereffect was measured using tones at the same frequency as the adaptation phase as well as at different frequencies. These studies have provided contradictory results. Specifically, in a first study [12], human subjects were exposed to an adaptation phase during which either 750 Hz or 3 kHz tones were presented concomitantly with a spatially disparate flash. At the end of adaptation, strong aftereffect was observed only using tones at the same frequency as the one used during the adaptation phase, whereas no aftereffect was observed using tones at the other frequency. Similar results were obtained in two subsequent studies on humans [15] and on monkeys [16] showing no or little transfer of aftereffect between 1 kHz and 4 kHz stimuli. The above-mentioned studies converged in suggesting that the aftereffect does not generalize over frequencies that are two octaves apart and hypothesized that the aftereffect may involve auditory neurons having sharp frequency tuning functions (i.e., neurons that respond only to a restricted range of sound frequencies) [12]. Nevertheless, another two studies are in heavy contrast with the previous ones, reporting aftereffect generalization not only across two-octave frequency range (750 Hz and 3 kHz) [17], but even across four-octave frequency range (400 Hz and 6.4 kHz) [18].

Some researchers [6, 17, 18] proposed that differences in the experimental design used in those studies (e.g., differences in the number of participants, in the size of audio-visual disparity, and in the magnitude of the resulting aftereffect) might have contributed in producing the discordant outcomes. However, these researchers too [6] stated that this apparent conflict remains largely unsolved. A potentially relevant aspect concerns the different sound intensities used across these studies: in particular, lower sound intensities were used in human studies reporting no aftereffect generalization (45 dB in [12] and 60 dB in [15]) compared to studies reporting aftereffect generalization (70 dB in [17] and 66 dB [18]). Indeed, neurophysiological data have shown that auditory stimulus intensity influences the response properties of single auditory neurons [20–24]. In particular, the spatial tuning function and the frequency tuning function are both affected by intensity and may be involved in aftereffect generalization. However, so far, this aspect has not been explicitly investigated. A better comprehension of the aftereffect generalization can have important implications as it may provide information on which neural mechanisms and structures—in particular which auditory cortical neurons—are involved in ventriloquism and in its related plastic effects and, more in general, may contribute to elucidate the processes of audiovisual interactions.

In the last decade, we have developed several neurocomputational models to investigate different aspects of organization and plasticity of multisensory integration, such as visual-auditory integration in the superior colliculus [25–29] and visual-tactile interaction in peripersonal space representation [30–32]. In particular, in a recent work [33], we proposed a simple model of visual-auditory integration in the cortex specifically devoted to simulation of ventriloquism effect and aftereffect. The model consisted of two one-dimensional layers of visual and auditory neurons communicating via intralayer and interlayer synapses, and employed Hebbian learning to reproduce the aftereffect phenomenon. However, this previous model exhibited a critical limit as the neurons in the model coded only for the spatial feature (i.e., the azimuthal position) of the stimuli, whereas the spectral characteristics of the auditory stimuli were neglected. Therefore, the model cannot be used to investigate the generalization of aftereffect across frequencies, nor to assess which neural mechanisms may explain the differences in aftereffect generalization observed in vivo.

The aim of this work is to develop an advanced version of the previous model in order to (i) mimic auditory neurons that code for both the spatial and spectral features of the stimulus, and that display response properties similar to biological neurons; (ii) reproduce ventriloquism effect and aftereffect in these new conditions; (iii) investigate the influence of different sound intensities on ventriloquism effect and aftereffect; (iv) provide a plausible interpretation of different extent of aftereffect generalization; (v) generate new predictions and suggest novel experiments to test model hypotheses.

## 2. Materials and Methods

The model is an extension of a previous one [33]. The previous model included two *one-dimensional* (1D) layers of visual and auditory neurons, communicating via reciprocal synapses. Each neuron in both layers coded for a particular azimuth of the external stimulus.

**Figure 1:**
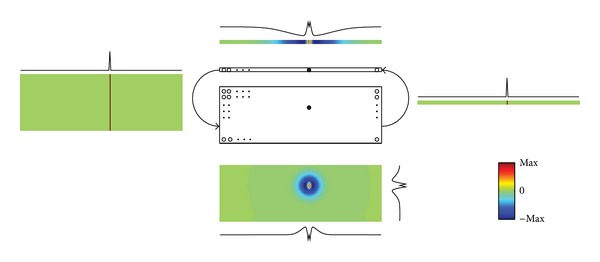
Network architecture. The two central rectangles represent the visual (array) and auditory (matrix) neurons. The other panels represent the basal connections that depart from the neurons marked with the two black bullets: the lateral panels represent interlayer connections, while the top and bottom panels represent intralayer connections. The colormaps are normalized to their maximum value and centered in 0.

In order to account for the spectral features of the auditory stimuli, the 1D layer of auditory neurons has been replaced by a *two-dimensional* (2D) matrix (Figure 1), where each neuron codes for a particular azimuth-frequency pair of the external auditory stimulus. The synapses have been changed accordingly. In the new version of the model, the visual layer has been maintained unchanged.

### 2.1. Model Description

The model consists of a 1D visual layer and of a 2D auditory layer. The 1D visual layer contains *N*_*p*_^*v*^ ( = 180) visual neurons. They code for the azimuth of the external visual stimulus and are spatiotopically aligned (proximal neurons code for proximal positions). The 2D auditory layer contains *N*_*p*_^*a*^ × *N*_*f*_^*a*^ ( = 180 × 40) auditory neurons. They code for a particular azimuth and a particular frequency of the external auditory stimulus and are spatiotopically and tonotopically aligned (proximal neurons code for proximal positions and proximal frequencies). Azimuths are linearly spaced by 1°, so the neurons cover 180° along the spatial dimension; frequencies are logarithmically spaced so the auditory neurons cover approximately eight octaves along the spectral dimension (one octave every five neurons).

Quantities referring to a generic neuron have superscripts indicating the neuron layer (*v*: visual layer; *a*: auditory layer) and subscripts indicating the neuron indices within the layer (one subscript for visual neurons in the 1D layer and two subscripts for the auditory neurons in the 2D layer). *u*(*t*) and *y*(*t*) are used to represent, respectively, the net input and the output of a given neuron at time *t*.

Neurons within each layer communicate via lateral intralayer synapses, and neurons in the two layers are reciprocally connected via cross modal interlayer synapses. Hence, the net input *u*(*t*) to a neuron is the sum of three contributions: an external input *e*(*t*); a lateral input *l*(*t*) (from other neurons in the same layer); a cross modal input *c*(*t*) (from neurons in the other layer). The activity *y*(*t*) of each neuron is computed by passing the net input *u*(*t*) through first-order dynamics and a steady-state sigmoidal relationship, with the saturation level set at 1 (i.e., the activity of each neuron is normalized to the maximum). Hence, for a visual neuron and an auditory neuron, the following differential equations can be written:

(1) $$\begin{array}{c} \tau_{y}\frac{dy_{i}^{v}\left(t\right)}{dt}=-y_{i}^{v}\left(t\right)+F\left(u_{i}^{v}\left(t\right)\right),\\ \tau_{y}\frac{dy_{ij}^{a}\left(t\right)}{dt}=-y_{ij}^{a}\left(t\right)+F\left(u_{ij}^{a}\left(t\right)\right), \end{array}$$

where *i* is the index of the visual neuron along the azimuth dimension, while *i* and *j* are the indices of the auditory neuron along the azimuth and the frequency dimensions, respectively. *τ*_*y*_ is the time constant, and *F*(*u*) represents a sigmoidal relationship

(2) $$\begin{array}{c} F\left(u\right)=\frac{1}{1+e^{-s(u-\theta )}} \end{array}$$

*s* and *θ* are parameters which establish the slope and the central position of the sigmoidal relationship, respectively. For the sake of simplicity, we used the same time constant and sigmoidal relationship for visual and auditory neurons (see parameters assignment).

In the following, each of the three inputs is described. It is worth noticing that, in order to avoid border effects, the layers have a circular structure. This means that the distance between two neurons at indices *i*_1_ and *i*_2_ along a dimension is always calculated as

(3) $$\begin{array}{c} d_{i1,i2}=\min\left(\left|i_{1}-i_{2}\right|,N-\left|i_{1}-i_{2}\right|\right), \end{array}$$

where *N* is the number of neurons along that dimension. To avoid complicating the notation, we will just write |*i*_1_ − *i*_2_| rather than *d*_*i*1,*i*2_ and (*i*_1_−*i*_2_)^2^ rather than (*d*_*i*1,*i*2_)^2^ in the following equations. 

(i)* The External Input e*(*t*). The visual external input is mimicked as a 1D Gaussian function, representing a spatially localized visual stimulus (e.g., a flash) filtered by the receptive fields (RFs) of the visual neurons in the 1D space map

(4) $$\begin{array}{c} e_{i}^{v}\left(t\right)=E_{0}^{v}\cdot \exp\left(-\frac{{\left(i-i_{p}^{v}\right)}^{2}}{2{\left(\sigma_{p}^{v}\right)}^{2}}\right), \end{array}$$

where *E*_0_^*v*^ is the stimulus intensity (arbitrary units), *i* is the index for a generic visual neuron, *i*_*p*_^*v*^ is the index at which the stimulus is centered, and *σ*_*p*_^*v*^ is related to the width of the visual RFs along the azimuth.

The auditory external input is reproduced as a 2D Gaussian function since it mimics an auditory stimulus localized in space and in frequency (tone) filtered by the RFs of the auditory neurons in the 2D space-frequency map

(5) $$\begin{array}{c} e_{ij}^{a}\left(t\right)=E_{0}^{a}\cdot \exp\left(-\frac{{\left(i-i_{p}^{a}\right)}^{2}}{2{\left(\sigma_{p}^{a}\right)}^{2}}-\frac{{\left(j-j_{f}^{a}\right)}^{2}}{{2\left(\sigma_{f}^{a}\right)}^{2}}\right) \end{array}$$

*E*_0_^*a*^ is the stimulus intensity (arbitrary units), *i* and *j* are the indices for a generic auditory neuron along the azimuth and frequency dimensions, respectively, *i*_*p*_^*a*^ and *j*_*f*_^*a*^ are the indices at which the stimulus is centered, and finally, *σ*_*p*_^*a*^ and *σ*_*f*_^*a*^ define the width of the auditory RFs along the two dimensions. To simulate the higher spatial resolution of the visual system, *σ*_*p*_^*v*^ is assumed smaller than *σ*_*p*_^*a*^ [33].

(ii) *The Lateral Input l*(*t*). This input originates from the lateral connections within each layer. We have

(6) $$\begin{array}{c} l_{i}^{v}\left(t\right)=\sum_{h}L_{i,h}^{v}\cdot y_{h}^{v}\left(t\right),\\ l_{ij}^{a}\left(t\right)=\sum_{h}\sum_{k}L_{ij,hk}^{a}\cdot y_{hk}^{a}\left(t\right) \end{array}$$

for the visual and auditory neurons, respectively, where *L*_*i*,*h*_^*v*^ is the synaptic strength from the presynaptic neuron at index *h* to the postsynaptic neuron at index *i* for the visual layer, while *L*_*ij*,*hk*_^*a*^ is the synaptic strength from the presynaptic neuron at index *hk* to the postsynaptic neuron at index *ij* for the auditory layer; *y*_*h*_^*v*^(*t*) and *y*_*hk*_^*a*^(*t*) represent the activity of the presynaptic neurons in the two layers. Lateral visual synapses are arranged according to a 1D Mexican hat, obtained as the difference of excitatory and inhibitory contributions each mimicked as a 1D Gaussian function

(7) Li,hv=Lex,i,hv−Lin,i,hv

(8) $$\begin{array}{c} L_{\text{ex},i,h}^{v}=L_{\text{ex}0}^{v}\cdot \exp\left(-\frac{{\left(i-h\right)}^{2}}{2{\left(\sigma_{\text{ex},p}^{v}\right)}^{2}}\right), \end{array}$$

(9) $$\begin{array}{c} L_{\text{in},i,h}^{v}=L_{\text{in}0}^{v}\cdot \exp\left(-\frac{{\left(i-h\right)}^{2}}{{2\left(\sigma_{\text{in},p}^{v}\right)}^{2}}\right), \end{array}$$

where *L*_ex0_^*v*^ and *L*_in0_^*v*^ define the strength of visual lateral excitation and inhibition; *σ*_ex,*p*_^*v*^ and *σ*_in,*p*_^*v*^ define their extension. In order to obtain a Mexican hat, excitation is stronger but narrower than inhibition (*L*_ex0_^*v*^ > *L*_in0_^*v*^; *σ*_ex,*p*_^*v*^ < *σ*_in,*p*_^*v*^).

Analogous relations hold for the auditory synapses but are generalized to the 2D case

(10) $$\begin{array}{c} L_{ij,hk}^{a}=L_{\text{ex},ij,hk}^{a}-L_{\text{in},ij,hk}^{a}, \end{array}$$

(11) $$\begin{array}{c} L_{\text{ex},ij,hk}^{a}=L_{\text{ex}0}^{a}\cdot \exp\left(-\frac{{\left(i-h\right)}^{2}}{{2\left(\sigma_{\text{ex},p}^{a}\right)}^{2}}-\frac{{\left(j-k\right)}^{2}}{{2\left(\sigma_{\text{ex},f}^{a}\right)}^{2}}\right), \end{array}$$

(12) $$\begin{array}{c} L_{\text{in},ij,hk}^{a}=L_{\text{in}0}^{a}\cdot \exp\left(-\frac{{\left(i-h\right)}^{2}}{{2\left(\sigma_{\text{in},p}^{a}\right)}^{2}}-\frac{{\left(j-k\right)}^{2}}{2{\left(\sigma_{\text{in},f}^{a}\right)}^{2}}\right), \end{array}$$

where *L*_ex0_^*a*^ and *L*_in0_^*a*^ define the strength of auditory lateral excitation and inhibition, and *σ*_ex,*p*_^*a*^ (*σ*_in,*p*_^*a*^) and *σ*_ex,*f*_^*a*^ (*σ*_in,*f*_^*a*^) define the extension of excitation (inhibition) along the azimuth and along the frequency dimension, respectively. In order to obtain a Mexican hat, we set *L*_ex0_^*a*^ > *L*_in0_^*a*^, *σ*_ex,*p*_^*a*^ < *σ*_in,*p*_^*a*^, and *σ*_ex,*f*_^*a*^ < *σ*_in,*f*_^*a*^.

Autoexcitation and autoinhibition are avoided in both layers:

(13) $$\begin{array}{c} L_{i,i}^{v}=0\;\forall i,\\ L_{ij,ij}^{a}=0\;\forall i,j. \end{array}$$

(iii)* The Cross Modal Input c*(*t*). This input originates from the interlayer synapses. We assume that a visual neuron at position *i* sends an excitatory synapse (*W*_*av*_) to all auditory neurons that code for the same azimuth (i.e., all auditory neurons along the *i*th column of the matrix) and receives an excitatory synapse (*W*_*va*_) from any of them. Hence, the cross modal inputs are computed as

(14) $$\begin{array}{c} c_{i}^{v}\left(t\right)=\sum_{j}W_{va}\cdot y_{ij}^{a}\left(t\right),\\ c_{ij}^{a}\left(t\right)=W_{av}\cdot y_{i}^{v}\left(t\right). \end{array}$$

It is worth noticing that we used just two parameters to represent all interlayer synapses, independently of the azimuth and of the auditory frequency.

### 2.2. Model Hebbian Rules

According to the previous paper [33], ventriloquism aftereffect may be explained assuming that, during exposure to a ventriloquism situation, lateral synapses within each layer can change according to Hebbian learning rules (adaptation phase).

In this paper, we adopted the same rules as in the previous one. In particular, in each layer, lateral excitatory and inhibitory synapses are subject to a potentiation Hebbian rule with a threshold for postsynaptic activity: excitatory synapses increase, whereas inhibitory synapses decrease in case of correlated pre- and postsynaptic activity, provided that postsynaptic activity overcomes a given threshold (*θ*_post_). Furthermore, two physiological constraints are imposed to the synapses: an individual saturation rule and a population normalization rule. In the following, only equations for the auditory layer are reported, as they have changed from 1D to 2D formulation.

For the auditory lateral synapses, we have

(15) $$\begin{array}{c} \Delta L_{\text{ex},ij,hk}^{a}\left(t\right)=\alpha_{\text{ex},ij,hk}^{a}\left(t\right)\cdot y_{hk}^{a}\left(t\right)\cdot {\left[y_{ij}^{a}\left(t\right)-\theta_{\text{post}}\right]}^{+},\\ \Delta L_{\text{in},ij,hk}^{a}\left(t\right)={-\alpha }_{\text{in},ij,hk}^{a}\left(t\right)\cdot y_{hk}^{a}\left(t\right)\cdot {\left[y_{ij}^{a}\left(t\right)-\theta_{\text{post}}\right]}^{+}, \end{array}$$

where *α*_ex,*ij*,*hk*_^*a*^(*t*) and *α*_in,*ij*,*hk*_^*a*^(*t*) are learning factors for the excitatory and inhibitory synapses, respectively, and [ ]^+^ denotes the function positive part, that is, [*x*]^+^ = max⁡⁡{*x*, 0}.

To accomplish the individual saturation rule, each excitatory synapse cannot overcome a maximum saturation value (*L*_max⁡_^*a*^). This is obtained assuming that the learning factor decreases with the synapse strength

(16) $$\begin{array}{c} \alpha_{\text{ex},ij,hk}^{a}\left(t\right)=\alpha_{\text{ex}0}\cdot \left(L_{\max}^{a}-L_{\text{ex},ij,hk}^{a}\left(t\right)\right), \end{array}$$

where *α*_ex0_ · *L*_max⁡_^*a*^ is the maximum learning factor (i.e., the learning factor when the synapse strength is zero). Similarly, each inhibitory synapse cannot become negative. Hence, the learning rate decreases with the synapse value

(17) $$\begin{array}{c} \alpha_{\text{in},ij,hk}^{a}\left(t\right)=\alpha_{\text{in}0}\cdot L_{\text{in},ij,hk}^{a}\left(t\right). \end{array}$$

To accomplish the population normalization rule, at each simulation step, all excitatory and inhibitory synapses entering a postsynaptic neuron are normalized, to maintain a constant synaptic input to each neuron. Normalization rules were applied separately for excitatory and inhibitory synapses. We have

(18) Lex,ij,hka(t+Δt) =Lex,ij,hka(t)+ΔLex,ij,hka(t)∑h=1Npa∑k=1Nfa(Lex,ij,hka(t)+ΔLex,ij,hka(t))  ·∑h=1Npa ∑k=1NfaLex,ij,hka(0),Lin,ij,hka(t+Δt) =Lin,ij,hka(t)+ΔLin,ij,hka(t)∑h=1Npa∑k=1Nfa(Lin,ij,hka(t)+ΔLin,ij,hka(t))  ·∑h=1Npa ∑k=1NfaLin,ij,hka(0),

where Δ*t* is the integration time step and Δ*L* denotes the synaptic change computed via (15)–(17) during a single integration step. Hence, if some of the entering excitatory synapses increase, others decrease; if some of the entering inhibitory synapses decrease, others increase.

Equations analogous to (15)–(18), with a 1D formulation, hold for the visual lateral synapses, and they can be found in our previous paper [33].

### 2.3. Parameters Assignment

All values for model parameters are reported in Table 1. All the standard deviations appearing in the equations are adimensional quantities as they represent measures of distance between the indices of the neurons within a given layer. External stimulus intensities (*E*_0_^*v*^ and *E*_0_^*a*^) are expressed in arbitrary units. The values for the intensity of the auditory external stimulus (*E*_0_^*a*^) are not reported in the table as it may assume different values in different simulations; hence, we will always specify the particular value adopted.

#### 2.3.1. Parameters of the Input-Output Relationship of Individual Neurons (*θ*, *s*, *τ*_*y*_)

According to our previous paper, parameters of the static sigmoidal relationship (central abscissa *θ* and slope *s*) and of the first-order dynamics (time constant *τ*_*y*_) of individual neurons were assumed equal for all neurons regardless of their respective layer. This choice was adopted in accordance with a parsimony principle, as ventriloquism effect and aftereffect can be explained without assuming any ad hoc differences in these parameters. Their values were taken from our previous paper [33]: *τ*_*y*_ is equal to 3 ms, in accordance with values usually adopted for neuron membranes (few milliseconds); *θ* and *s* were set so that the neuron remains almost silent in absence of the input and exhibits a smooth transition from silence to saturation as the input increases.

#### 2.3.2. Parameters Characterizing Visual Neurons (*E*_0_^*v*^, *σ*_*p*_^*v*^, *L*_ex0_^*v*^, *L*_in0_^*v*^, *σ*_ex,*p*_^*v*^, *σ*_in,*p*_^*v*^, *W*_*av*_)

The value of the standard deviation of the external visual stimulus (*σ*_*p*_^*v*^ representing the width of the visual RF along the azimuth) and the values of the lateral synapses parameters in the visual layer (*L*_ex0_^*v*^, *L*_in0_^*v*^, *σ*_ex,*p*_^*v*^, *σ*_in,*p*_^*v*^) were taken from our previous paper, since the visual layer was not changed. On the overall, the assigned values warrant that a unimodal visual stimulus produces a narrow activation in the visual area, in agreement with the high spatial acuity of the visual system. In particular, *σ*_*p*_^*v*^ was set equal to just few units (= 4). In this work, the intensity of the visual stimulus *E*_0_^*v*^ was fixed at value 15 in all the simulations; this value produces a quite elevated activation in the visual area, so as to mimic a well-perceivable and well-localizable visual input, as it is generally used in the in vivo studies to produce the ventriloquism effect [5, 7, 8].

The value of the cross modal synapses *W*_*av*_ from the visual neurons to the auditory neurons was assigned (i) to be sufficiently low so that a unimodal visual stimulus does not induce any phantom activation in the auditory layer and (ii) to be sufficiently high so that a visual stimulus can reinforce the activation of auditory neurons when they are just a little above the silence state. The value for these synapses was changed with respect to the previous paper since the auditory layer was modified.

**Figure 2:**
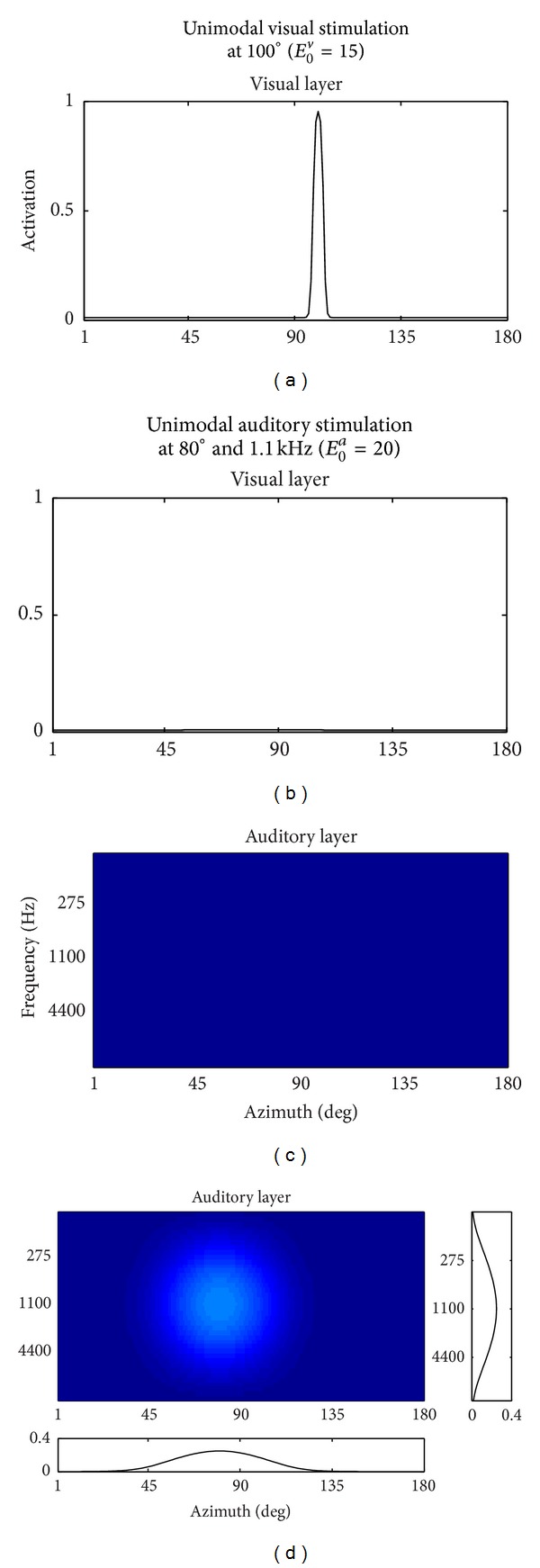
Network response to unimodal stimuli. Left panels ((a) and (c)) show the response of the visual and auditory layers to a visual stimulus at 100° with *E*_0_^*v*^ = 15. Right panels ((b) and (d)) show the response of the visual and auditory layers to an auditory stimulus at 80° and 1.1 kHz with *E*_0_^*a*^ = 20. The two insets in (d) display the response profiles along the azimuth at frequency 1.1 kHz (bottom inset) and along the frequency at azimuth 80° (right inset).

These aspects are summarized in Figures 2(a) and 2(c). They show the network response in steady-state condition (i.e., after the transient was exhausted) to a unimodal visual stimulus applied at position 100° and with intensity *E*_0_^*v*^ = 15. The stimulus was maintained throughout the overall simulation. Activation of the visual neurons assumes high values (close to saturation level) only nearby the position of the stimulus and declines sharply to zero as moving away from it, thus signaling a well-localizable visual stimulus. No activation is produced in the nonstimulated auditory layer.

#### 2.3.3. Parameters Characterizing Auditory Neurons (*σ*_*p*_^*a*^, *σ*_*f*_^*a*^,  *L*_ex0_^*a*^, *L*_in0_^*a*^, *σ*_ex,*p*_^*a*^, *σ*_in,*p*_^*a*^, *σ*_ex,*f*_^*a*^, *σ*_in,*f*_^*a*^, *W*_*va*_)

The values for the standard deviations of the external auditory stimulus (*σ*_*p*_^*a*^ and *σ*_*f*_^*a*^ representing the width of the auditory RF along azimuth and frequency) and for the lateral synapses parameters in the auditory layer (*L*_ex0_^*a*^, *L*_in0_^*a*^, *σ*_ex,*p*_^*a*^, *σ*_in,*p*_^*a*^, *σ*_ex,*f*_^*a*^, *σ*_in,*f*_^*a*^) were assigned in order to satisfy the following requirements.

- *σ*_*p*_^*a*^ was assumed larger than *σ*_*p*_^*v*^, to reproduce the lower spatial acuity of the auditory system with respect to the visual one.
- The balance between lateral excitation and inhibition was set to prevent an excessive activation spreading across all the auditory neurons.
- A single unimodal auditory stimulus applied at a specific azimuth and frequency produces an activation in the auditory layer that declines from the peak value to zero moving by some tenths of degrees along the azimuth and by some octaves along the frequency. An example of this response is shown in Figures 2(b) and 2(d) that display the network response (at steady-state condition) to an auditory stimulus applied at 80° and frequency 1.1 kHz, with intensity *E*_0_^*a*^ = 20 (the stimulus was maintained throughout the overall simulation). Auditory activation assumes low values and has a wide extension, involving several neurons both along the azimuth and the frequency. In particular, the bottom inset shows the horizontal profile (at frequency 1.1 kHz) of the 2D activation map, and the right inset shows the vertical profile (at 80°) of the 2D activation map. Due to the symmetry of the network in basal conditions (i.e., before adaptation), the bottom profile corresponds to the azimuthal tuning function of the neuron at 80°, 1.1 kHz (i.e., the neuron response to identical stimuli placed at all the 180 azimuth positions and at the fixed frequency of 1.1 kHz), and the right profile corresponds to the frequency tuning function of the same neuron (i.e., the neuron response to identical stimuli placed at all the frequencies and at the fixed azimuth position of 80°). These tuning functions are representative of the behavior of all neurons in basal conditions: the azimuth tuning function is not flat but exhibits a response over a confined region spanning by 60°–70°, and the frequency tuning function is quite broad, larger than 1-2 octaves. Biological neurons showing these properties have been found both in the primary auditory cortex and, to a greater proportion, in the caudomedial field [20, 21, 23, 24]. It is worth noticing that in Results section a sensitivity analysis is presented, assessing the effect of the auditory stimulus intensity on the azimuth and frequency tuning functions.

The value of the cross modal synapses *W*_*va*_ from the auditory neurons to the visual neurons was assigned according to the same principles as *W*_*av*_. Furthermore, *W*_*va*_ was set ≅*W*_*av*_/*N*_*f*_^*a*^, so that the action exerted by the auditory neurons on visual neurons (each single visual neuron is targeted by all *N*_*f*_^*a*^ auditory neurons at the same spatial position) is comparable with the action exerted by the visual neurons on auditory neurons (each single auditory neuron is targeted by the single visual neuron at the same spatial position).  Figure 2(b) indicates that the unimodal auditory stimulus does not induce any phantom activation in the visual layer.

#### 2.3.4. Parameters Characterizing Hebbian Rules (*α*_in0_, *α*_ex0_, *θ*_post_, *L*_max⁡_^*a*^, *L*_max⁡_^*v*^)

Hebbian rules adopted in this paper are the same as in the previous one and their parameters were assigned according to the same principles as in the previous paper. Values for the learning rates of the inhibitory and excitatory synapses (parameters *α*_in0_ and *α*_ex0_) were set so that synapses were gradually modified during training and reached a new steady-state pattern within 1000 updating steps. In agreement with our previous paper [33], the value of the postsynaptic threshold (*θ*_post_) was set = 0.5, so that changes occurred only for synapses targeting neurons activated above 50% of their maximal activation. This value warrants that reciprocal synapses between two activated neurons modify asymmetrically. Finally, as in the previous paper, the maximum saturation value for each excitatory synapse in the auditory and visual layer (*L*_max⁡_^*a*^ and *L*_max⁡_^*v*^) has been assumed equal to the corresponding maximum strength in basal conditions (*L*_ex0_^*a*^ in (11) and *L*_ex0_^*v*^ in (8)).

### 2.4. Simulation Description and Evaluation of Network Performances

Equations in the model were numerically solved within the software environment MATLAB (The MathWorks, Inc.), using the Euler integration method, with an integration step sufficiently small to avoid instability and to warrant convergence.

Adaptation phases were mimicked by exposing the untrained network (basal values for the synapses) to a spatially discrepant visual-auditory stimulation—starting from network null condition (i.e., zero activity for all neurons)—and maintaining the stimuli for 1000 steps. During the overall length of the simulation (1000 steps), the lateral synapses within the two layers were trained according to the Hebbian rules.

Simulations were performed to assess network behavior before adaptation (basal conditions) and after adaptation (trained network). In all these simulations, external stimuli were delivered to the network and maintained until the network reached a new steady-state condition, at which neuron responses and network behavior were evaluated. In the psychophysical literature, ventriloquism effect and aftereffect are assessed by measuring the discrepancy between the perceived sound location and the actual sound location (perceptual shift). Hence, to evaluate network behavior in terms of ventriloquism effect and aftereffect, we need to compute a quantity representing the perceived stimulus location from the activity *y*(*t*) of all neurons within a layer. Here, we used the barycenter metric [33]: the perceived location is taken as the average position (barycenter) of the layer population activity. Hence, the perceived location of an auditory stimulus is computed as follows:

(19) $$\begin{array}{c} z^{a}\left(t\right)=\frac{\sum_{i}\sum_{j}\left(y_{ij}^{a}\left(t\right)\cdot i\right)}{\sum_{i}\sum_{j}y_{ij}^{a}\left(t\right)}, \end{array}$$

and the perceived location of a visual stimulus is computed as follows:

(20) $$\begin{array}{c} z^{v}\left(t\right)=\frac{\sum_{i}\left(y_{i}^{v}\left(t\right)\cdot i\right)}{\sum_{i}y_{i}^{v}\left(t\right)}, \end{array}$$

where *t* is a generic time instant after the network has reached the new steady-state condition.

## 3. Results

This section shows the effects of different sound intensities on network behavior. First, we analyzed how the sound intensity affects the response to unimodal auditory stimulation. Then, we analyzed how the sound intensity influences the ventriloquism effect, that is, the shift in sound location during the presentation of a spatially disparate visual-auditory stimulation. Subsequently, adaptation phases were simulated: ventriloquism aftereffect and its generalization across frequencies were investigated as a function of the sound intensity used during the adaptation phase and during the testing phase.

### 3.1. Response to Unimodal Auditory Stimulus

A sensitivity analysis was performed to investigate how the intensity of the auditory stimulus modulates the response properties of the neurons: the azimuthal tuning function and the frequency tuning function of the auditory neurons were evaluated in basal conditions (before adaptation) at different intensities of the auditory stimulus (*E*_0_^*a*^).

**Figure 3:**
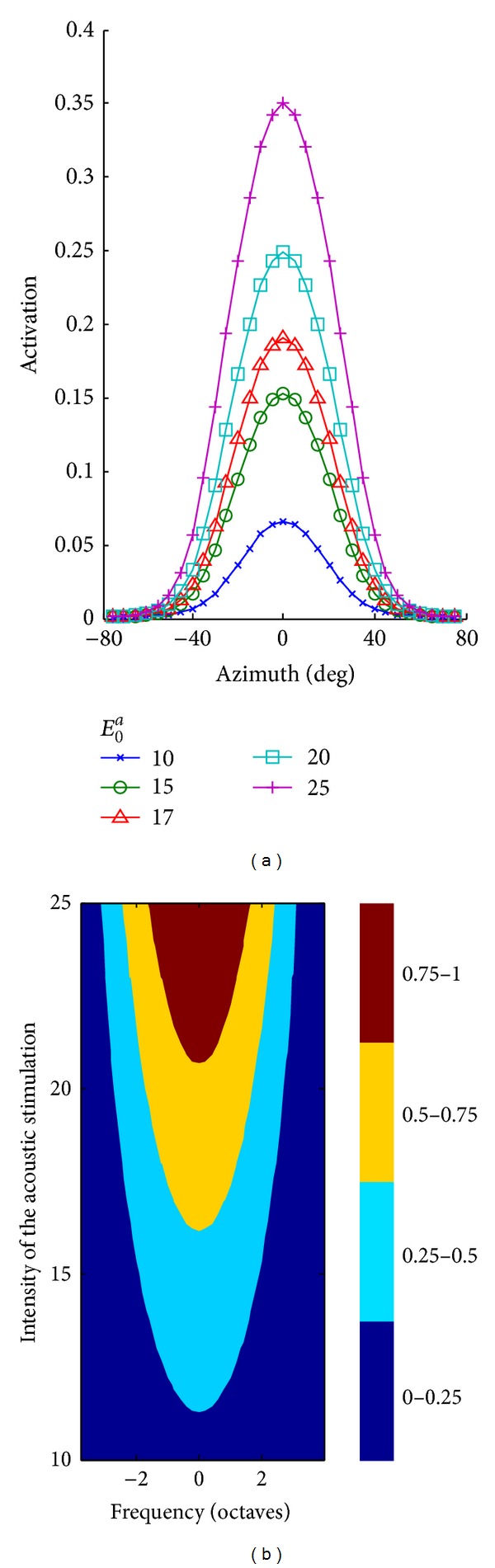
Sensitivity analysis on the tuning functions of auditory neurons. (a) shows the azimuthal tuning function of a generic auditory neuron for different intensities of the auditory stimulus. (b) shows the frequency tuning function of a generic auditory neuron for different intensities of the auditory stimulus (along the *y* axis); the map is normalized with respect to the peak activation.

**Figure 4:**
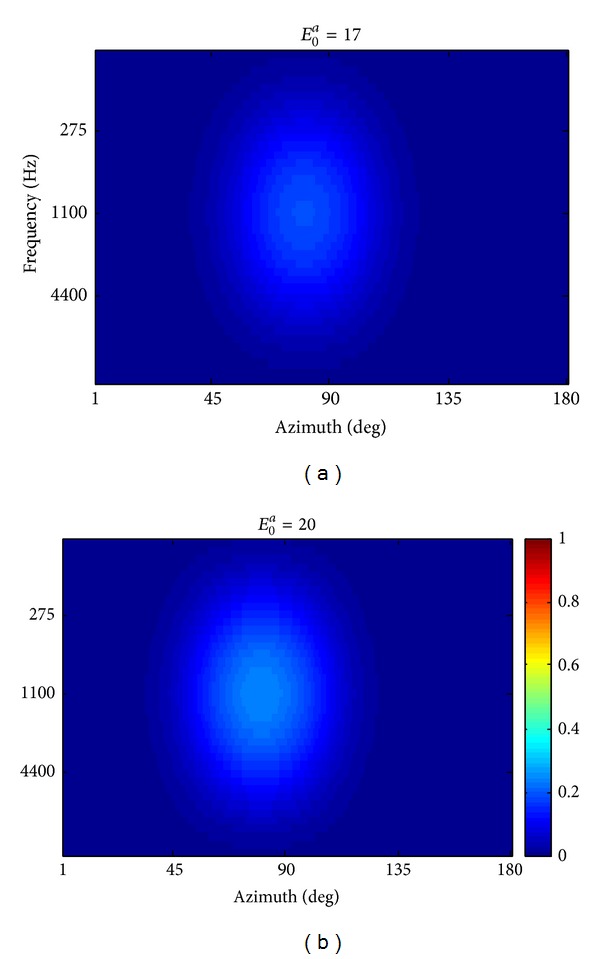
Activation of auditory neurons in response to an auditory stimulus applied at 80° and 1.1 kHz with intensity *E*_0_^*a*^ = 17 (a) and *E*_0_^*a*^ = 20 (b).

Figure 3(a) displays the results as to the azimuthal tuning function. As the stimulus intensity increased, the shape of the function was maintained, while its peak clearly grew. The width of the function—evaluated as the half-maximum width (i.e., the azimuthal range from the maximum response to one-half the maximum response)—slightly increased, expanding by *≈* 8° when stimulus intensity shifts from 10 to 25. These model results are in agreement with data found in some neurophysiological works: an increase in the width and in the peak of the azimuthal response, with increasing sound intensity, was observed in certain populations of auditory neurons both in the primary auditory cortex (see e.g., Figures 1, 4, and 5 in [20]) and in the caudomedial field (see e.g., Figures 4 and 12 in [34]).

Figure 3(b) displays the results as to the frequency tuning function. We adopted the same representation as usually adopted in neurophysiological studies: the response of the neuron is represented by a colormap as a function of the stimulus frequency and intensity (hence, each row of the figure corresponds to the frequency tuning function at the respective stimulus intensity). In this representation—sometimes termed frequency response area (FRA) [21, 22]—the neuron response is normalized to the peak, and the colormap is subdivided into discrete levels to depict regions eliciting a response <25%, between the 25% and the 50%, between the 50% and 75%, and >75%. The mimicked auditory neurons presented narrower and lower frequency tuning functions at small stimuli intensities and broader and higher tuning functions at larger stimuli intensities. These model results match the behavior of some real cortical auditory neurons especially in the caudomedial field (see e.g., Figure 3 in [21] and Figure 1 in [22]).

According to these model results, an increase in auditory stimulus intensity induced a higher and broader activation in the auditory layer, as depicted in Figure 4.

### 3.2. Ventriloquism Effect

**Figure 5:**
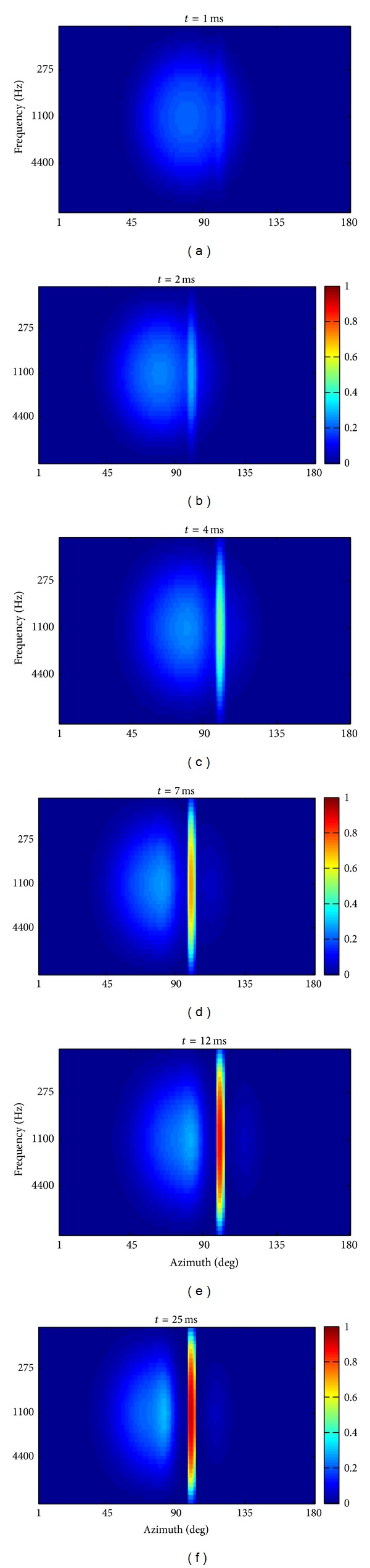
Activation of auditory neurons at different simulation steps (*t* = 1, 2, 4, 7, 12, and 25 ms) during the presentation of a visual stimulus at 100° and an auditory stimulus at 80° and 1.1 kHz (*E*_0_^*a*^ = 20).

In order to clarify the origin of the ventriloquism effect, Figure 5 shows different snapshots of the auditory layer activation at different simulation steps during the simultaneous presentation of a visual stimulus at 100° and an auditory stimulus at 80° and 1.1 kHz, with intensity *E*_0_^*a*^ = 20. Immediately at the beginning of the stimulation, a large portion of the auditory layer was activated involving also neurons at position 100° (Figure 5(a)), where the visual stimulus was applied. Hence, a positive feedback occurred between visual and auditory neurons at 100° thanks to the interlayer synapses; as a consequence, a higher activation began to emerge in the auditory area at that position (Figure 5(b)). Auditory neurons around 100° became rapidly more active (Figures 5(c) and 5(d)); they further reinforced their activation via reciprocal lateral excitation and via the interlayer positive feedback and competed with more distant neurons (at 10°–15° distance) via lateral inhibition (Figure 5(e)). At steady state (Figure 5(f)), the auditory activation was characterized by an elongated strip of strongly active neurons close to 100° and a broad area of less active neurons left of 90°. This activation resulted in a perceptual shift—perceived location minus actual location—of 4° (ventriloquism effect). On the contrary, the perceptual shift of the visual stimulus towards the sound location was just negligible (less than 0.1°).

**Figure 6:**
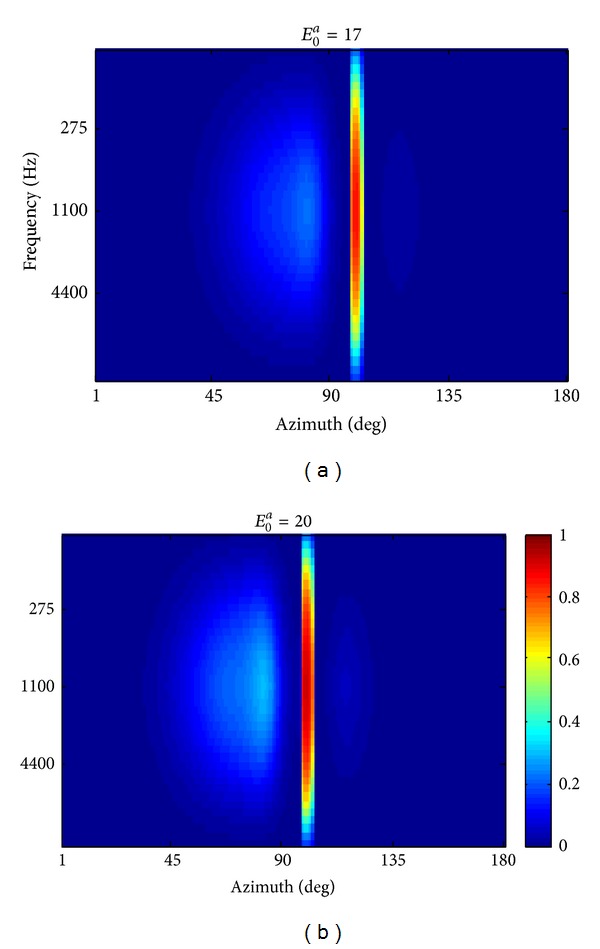
Activation of auditory neurons (at steady state) in response to spatially disparate visual auditory stimulation (the same stimuli position as in Figure 5) using auditory stimulus intensity *E*_0_^*a*^ = 17 (a) and *E*_0_^*a*^ = 20 (b).

Different sound intensities may produce different ventriloquism effects, due to the different activation in the auditory area.  Figure 6(a) shows the steady-state response of the auditory layer to a spatially disparate visual-auditory stimulation as in Figure 5 (that is the visual stimulus was applied at 100° and the auditory stimulus was applied at 80° and 1.1 kHz), using an intensity of 17 (*E*_0_^*a*^ = 17), instead of 20, for the auditory stimulus. To facilitate comparison, the last panel of Figure 5 is reported in Figure 6(b) too. In case of higher auditory stimulus intensity, (cf. Figures 6(a) and 6(b)) the auditory neurons are on the overall more activated. It is interesting to note that the amount of perceptual sound shift was lower in case of the higher stimulus intensity (3.6° at *E*_0_^*a*^ = 20 versus 4.7° at *E*_0_^*a*^ = 17): this was due to the higher and wider activation in the region left of 90° that strongly influenced the barycenter computation.

**Figure 7:**
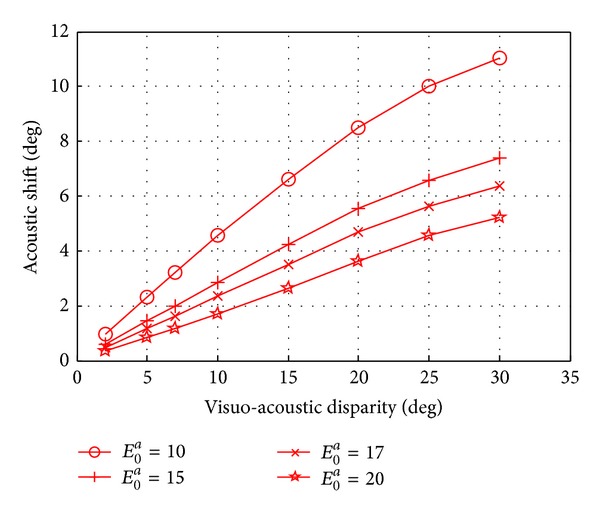
Ventriloquism effect simulated using different sound intensities and displayed as a function of the spatial disparities between the visual and the auditory stimuli.

Values of ventriloquism effects at different sound intensities are reported in Figure 7 as a function of the visual-auditory spatial disparity. Patterns of ventriloquism effect displayed in Figure 7 are in line with in vivo results [5, 10, 35–38]: in particular the model reproduces the increase in the magnitude of the effect as the visual-auditory spatial disparity increases and as the saliency of the visual stimulus increases with respect to the auditory one. In all the cases, the perceptual shift of the visual stimulus obtained with the model was below 0.5°, according to behavioral data [5].

For the sake of simplicity, we did not train the lateral synapses when investigating the ventriloquism effect. In this way, we considered only the sound shift due to the conflicting audiovisual input and neglected the additional effect due to synaptic adaptation.

### 3.3. Ventriloquism Aftereffect and Its Generalization across Frequencies

During each adaptation phase, the visual stimulus was applied at azimuth 100°, whereas the auditory stimulus was placed at azimuth 80° and at frequency 1.1 kHz (i.e., the same conditions as in Figures 5 and 6), and lateral synapses within each layer were trained. Hence, 1.1 kHz represented the adaptation frequency and 80° represented the adaptation position. We simulated seven adaptation phases, each differing from the other as to the intensity of the auditory stimulus, which ranged from 17 to 23. After each adaptation phase, the network was tested to assess the perceptual shift due to ventriloquism aftereffect and its generalization over frequency, by applying an auditory stimulus (test stimulus) at the adaptation position and either at the adaptation frequency or at a different frequency. In the following, we will denote the intensity of the auditory adaptation stimulus as *E*_0_^*a*^_adapt_ and the intensity of the test stimulus as *E*_0_^*a*^_test_. First, after each adaptation phase, the trained network was tested with *E*_0_^*a*^_test_ = *E*_0_^*a*^_adapt_, as usually done in psychophysical studies [12, 15, 17, 18].

**Figure 8:**
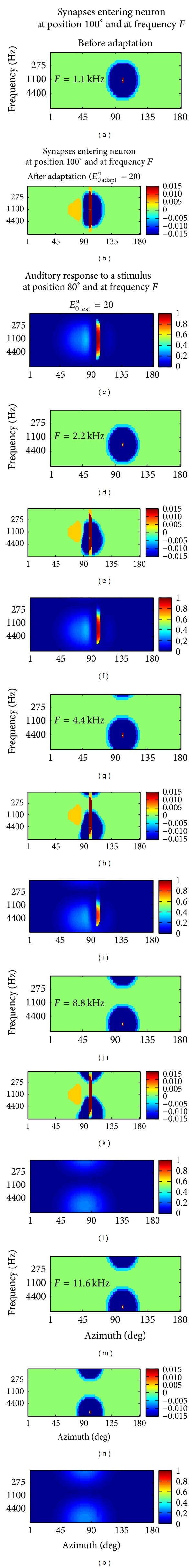
Effects of network training with *E*_0_^*a*^_adapt_ = 20 and of network testing (after adaptation) with *E*_0_^*a*^_test_ = 20. The adaptation position was 80°, and the adaptation frequency was 1.1 kHz. The first column shows the untrained synapses that target neuron at azimuth 100° and frequency *F*; the second column shows the same synapses after the adaptation; the third column shows the response to the test stimulus applied at azimuth 80° and frequency *F*. The value for the frequency *F* depends on the row of panels, as indicated inside the left panel in each row.

**Figure 9:**
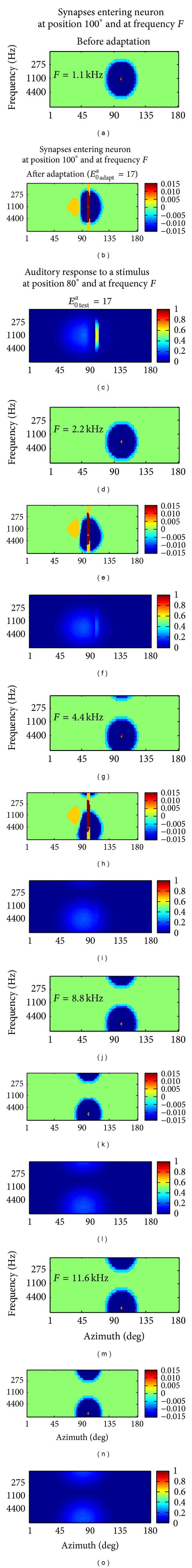
Effects of network training with *E*_0_^*a*^_adapt_ = 17 and of network testing (after adaptation) with *E*_0_^*a*^_test_ = 17. The adaptation position was 80° and the adaptation frequency was 1.1 kHz. The panels show the same quantities as respective panels in Figure 8.

Figure 8 shows the results of the network training with *E*_0_^*a*^_adapt_ = 20 and network testing with *E*_0_^*a*^_test_ = 20; Figure 9 displays the results of the network training with *E*_0_^*a*^_adapt_ = 17 and network testing with *E*_0_^*a*^_test_ = 17. In both figures, the first two columns represent the synapses before adaptation (first column) and after adaptation (second column) targeting five different auditory neurons, all located at 100° (the position of the visual stimulus during adaptation) and at five different frequencies (1.1 kHz, 2.2 kHz, 4.4 kHz, 8.8 kHz, and 11.6 kHz). The third column displays the response of the trained network to the test stimulus applied at the adaptation position (80°) and at the five different frequencies.

The comparison of the two figures highlights that a higher *E*_0_^*a*^_adapt_ induced a greater reinforcement of excitatory synapses over a wider frequency range. As an example, the neuron at three octaves apart from the adaptation frequency exhibited synapses modification only in case of *E*_0_^*a*^_adapt_ = 20 (compare Figures 8(k) and 9(k)). Furthermore, network response to test stimuli displayed a clear occurrence of aftereffect in (c), (f), and (i) (i.e., across two octaves) of Figure 8, while it displayed only a mild aftereffect just in (a) (i.e., at the adaptation frequency) of Figure 9.

**Figure 10:**
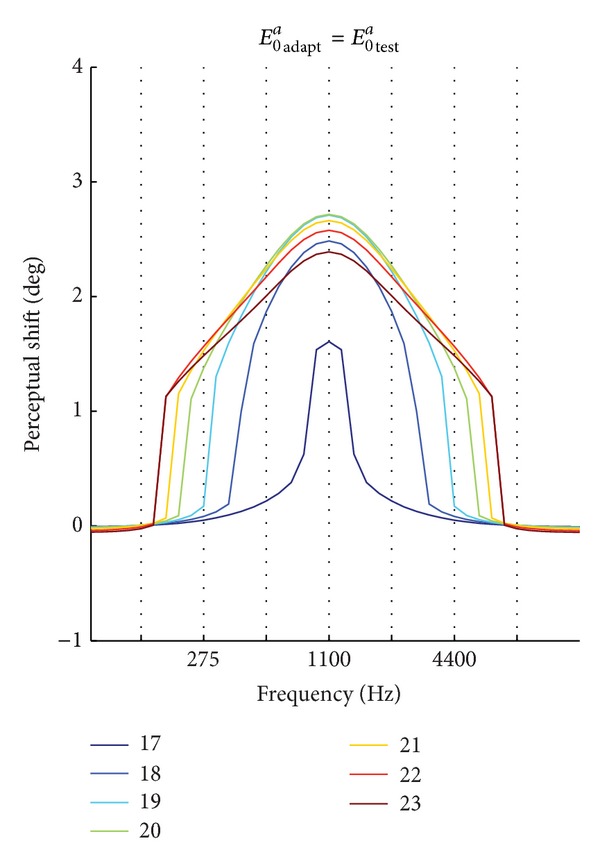
Aftereffect generalization. The plot shows the influence of the auditory stimulus intensity on the aftereffect generalization across frequencies (one octave per grid line), when the adaptation intensity is equal to the test intensity (*E*_0_^*a*^_adapt_ = *E*_0_^*a*^_test_).

Figure 10 shows the values of the aftereffect obtained by varying the test stimulus over the whole range of frequencies, after each adaptation phase (using *E*_0_^*a*^_adapt_ = *E*_0_^*a*^_test_). At a low sound intensity (= 17), a slight aftereffect (1.61°) occurred only at the adaptation frequency (see also Figure 9). As intensity increased (= 18), a stronger aftereffect occurred at the adaptation frequency (*≈* 2.49°) and aftereffect is generalized approximately across one-octave range. At further higher intensity (= 19), aftereffect is transferred over a wider range of frequencies, although aftereffect was reduced to zero at two-octave distance (4.4 kHz). The latter two conditions qualitatively agree with results of the two studies [12, 15] reporting significant aftereffect at the adaptation frequency and no transfer of aftereffect across sounds that differed by two octaves. As intensity of the auditory stimulus further increased (>19), aftereffect transferred to a larger range of frequencies showing a significant amount still at two-octave distance and even generalizing across almost three octaves (for intensities as high as 22 and 23), although aftereffect at these distances was lower than at the adaptation frequency. Simulated patterns of aftereffect at high intensities (≥20) are in qualitative agreement with those observed in the studies by Frissen et al. [17, 18] reporting aftereffect generalization across two and even more octaves and observing a decreasing gradient as moving away from the adaptation frequency.

**Figure 11:**
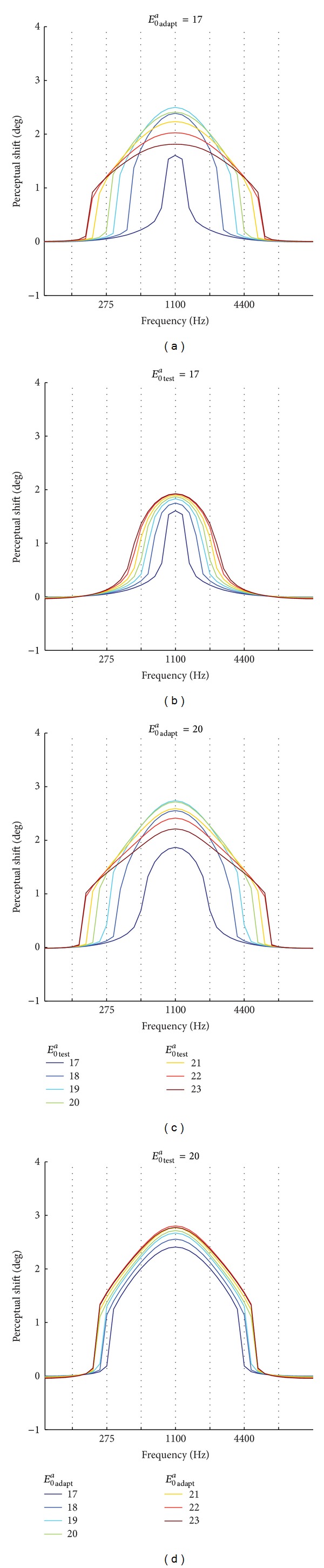
Aftereffect generalization when using different values of intensity for the adaption stimulus (*E*_0_^*a*^_adapt_) and for the test stimulus (*E*_0_^*a*^_test_). In the left panels, *E*_0_^*a*^_adapt_ was kept fixed (*E*_0_^*a*^_adapt_= 17 in (a) and *E*_0_^*a*^_adapt_ = 20 in (c)), while *E*_0_^*a*^_test_ ranged from 17 to 23 (the meaning of the line color is indicated in the legend). In the right panels, *E*_0_^*a*^_test_ was kept constant (*E*_0_^*a*^_test_ = 17 in (b) and *E*_0_^*a*^_test_ = 20 in (d)) to test the network trained with *E*_0_^*a*^_adapt_ ranging from 17 to 23 (the meaning of the line color is indicated in the legend).

It is worth noticing that model results shown in Figures 8–10 were obtained with the test stimulus having the same intensity as the adaptation one (as done in in vivo studies). Hence, two joined factors contributed to the aftereffect generalization: the different amount of synapses modifications (due to the different adaptation intensities) and the different intensities of the test stimulus. In order to discern the role of these two factors, we performed two additional sets of simulations: (i) the network trained at a given adaptation intensity was tested at each of the test intensities (ranging from 17 to 23); (ii) a given test intensity was used to test the network trained at each of the adaptation intensities (ranging from 17 to 23). Exemplary results are reported in Figure 11. The model predicts that the intensity of the test stimulus plays a crucial role in determining the range of frequencies over which aftereffect is generalized (Figures 11(a) and 11(c)). At *E*_0_^*a*^_adapt_ = 17 (a), the network behavior shifted from no generalization to generalization across more than two octaves, when *E*_0_^*a*^_test_ shifted from 17 to 23. At *E*_0_^*a*^_adapt_ = 20 (c), a significant shrinking of generalization occurred when *E*_0_^*a*^_test_ decreased down to 17.

According to model results, the adaptation intensity (Figures 11(b) and 11(d)) contributed to increase the magnitude of aftereffect but influenced only mildly the range of frequencies over which aftereffect occurred. At *E*_0_^*a*^_test_ = 17 (b), generalization remained below two-octave distance even at the highest *E*_0_^*a*^_adapt_ (= 23). At *E*_0_^*a*^_test_ = 20 (d), generalization is only scarcely influenced by *E*_0_^*a*^_adapt_.

Results displayed in Figure 11 may represent testable predictions generated by the model that can be verified by new experimental studies.

## 4. Discussion

In this work, we provided an extended version of our previous model [33] to explore generalization of aftereffect across sound frequencies and to provide a possible interpretation of the opposing results presented in the literature. Model results indicate that the intensity of the auditory stimulus, affecting the amount of activation in the auditory area, strongly influences aftereffect generalization across frequencies (Figures 8–11). In particular, adaptation stimuli at higher intensities cause a stronger synaptic reinforcement, while test stimuli at higher intensities, even far from the adaptation frequency, can easily boost the synapses trained during the adaptation phase. Hence, intensities of both the adaptation and test stimuli concur in establishing the magnitude of the aftereffect and the extension of its generalization: the intensity of the test stimulus has a preeminent role in determining the frequency range over which aftereffect is generalized; the intensity of the adaptation stimulus mainly contributes in increasing the magnitude of the produced aftereffect.

Model structure and results have several correspondences with neurophysiological and psychophysical data. The properties of auditory neurons in the model (azimuth sensitive range as large as 60°–70° and tuned in frequency, expanding their areas of response as stimulus intensity increases) match those of some biological neurons. Populations of azimuth sensitive neurons were found in the primary auditory cortex (A1) and, in greater proportion, in the caudomedial field (CM) [23, 34]. Such neurons usually have azimuth tuning functions that encompass a quadrant or almost a hemifield of space; with increasing sound levels, azimuth tuning functions assume higher values and expand (anyway, remaining confined to a hemifield of space). Neurons tuned in frequency were observed both in A1 and in CM; especially CM neurons exhibit spectral tuning functions modulated by the stimulus intensity, with narrower tuning bandwidth at lower stimulus intensities and broader tuning bandwidth at higher stimulus intensities [21, 24]. Moreover, auditory neurons in the model are spatiotopically and tonotopically organized. Whereas the assumption of the tonotopic organization is in agreement with the physiological knowledge (tonotopic organization was found both in area A1 and CM [21, 24]), a clear topographical representation of space has not been demonstrated in the auditory cortex [23]. However, the spatial topography adopted in the model should be intended mainly as functional rather than anatomical. That is, we intended that neurons coding for proximal azimuths—not necessarily proximal in the anatomical space—tend to excite reciprocally and to show correlated activities (i.e., they are functionally proximal), whereas, neurons coding for distant azimuths—not necessarily distant in the anatomical space—tend to inhibit reciprocally and to show uncorrelated activities (i.e., they are functionally distant). In our model, functionally proximal (distant) neurons were set at adjacent (remote) locations to simplify numerical implementation.

Auditory neurons in the model receive direct connections from the visual layer. The latter may represent the primary visual area. Indeed, some recent works [10, 11] show that damages to the primary visual area abolished ventriloquism. Furthermore, several studies indicate the existence of direct connections among putatively unimodal areas (such as visual and auditory) at an early processing stage [39–41]; direct visual influences on the auditory cortex were localized, in particular, in the caudomedial and caudolateral fields [42].

With such structure and organization, the ventriloquism effect simulated with the model (Figure 7) shows patterns consistent with those observed in vivo. Moreover, to reproduce ventriloquism aftereffect, Hebbian rules were adopted to modify intralayer lateral synapses. In agreement with our previous paper [33], we adopted postgating rules with local constraint (individual saturation) and global constraint (normalization). These rules are biologically plausible [43, 44] and were already proven to be suitable to reproduce ventriloquism aftereffect [33].

By using these Hebbian rules and by changing the intensity of the auditory stimulus (Figures 8–10), different extent of aftereffect generalization across frequencies is obtained with the model, ranging from no generalization (at lower stimuli intensity) to more than two-octave generalization (at higher stimulus intensity). These results are in agreement with in vivo studies reporting no generalization at lower sound intensities (45 dB and 60 dB) [12, 15] and generalization across two or even more octaves at higher sound intensities (70 and 66 dB) [17, 18].

An important point that deserves some comments concerns the small range of sound levels across which opposite behaviors are experimentally observed: no generalization across frequencies at 60 dB [15] and wide generalization at 66 dB [18]. A possible interpretation is that these studies were performed close to a critical sound level at which transition from no generalization to generalization occurs. Although so far this aspect cannot be clearly established, some speculations can be drawn, also inspired by model hypothesis and results. In particular, the model suggests that different extent of aftereffect generalization may result from different sound intensities that induce different amounts of activation in the auditory area. This is supported by physiological data, according to which spectral and spatial tuning functions of auditory neurons (both in AI and CM) rise and enlarge as stimulus intensity increases [21, 22, 24, 34]. Despite interneuron variability, the spectral tuning functions of several exemplary neurons in A1 and especially in CM seem to remain confined within about one or two octaves at sound levels below 60 dB and to exhibit a prominent enlargement right between 60 dB and 70 dB sound level (see, e.g., figures from 3 to 6 in [21]; see also Figures 1 and 2 in [22]). Another group of researchers [24] observed that the bandwidth of CM neurons extensively enlarged (encompassing even 5 and 6 octaves) especially for sound levels above 60 dB. Furthermore, the spatial tuning function of CM neurons exhibited almost indistinguishable patterns at three tested lower sound intensities (25, 35, and 55 dB), whereas it significantly widened and increased at sound level of 75 dB (see Figure 12 in [34]). All these data might suggest that there is a small range of sound levels (between 60 dB and 70 dB) across which the activation of auditory neurons changes significantly (shifting from a narrow and low activation to a broad and high activation) and can induce substantially different behavioral responses (from no generalization to wide generalization of aftereffect). Moreover, also the model predicts the existence of a small interval of sound intensities that leads to largely different extent of aftereffect generalization across frequencies (Figure 10): no generalization is observed at sound intensity of 17; one-octave generalization occurs at 18 and 19; two-octave generalization occurs at 20; almost three-octave generalization is obtained at sound intensities above 20. Although it is not possible to establish a definite correspondence between model sound intensities and physical sound levels, we can speculate that model intensity below 20 may roughly correspond to physical sound levels lower than or equal to 60 dB, and model sound intensity above 20 may correspond to physical sound levels above 65 and 66 dB.

However, all previous speculations are still far from being conclusive, and further experimental studies are necessary to thoroughly test the hypothesis (suggested by in vivo data and by model results) that a small range of sound levels allows transition from no generalization to generalization across frequencies. In particular, psychophysical studies should be performed to investigate systematically aftereffect generalization across frequencies when the sound level is varied at small step (e.g., 1 dB step), within the limited range between 60 dB and 70 dB.

Finally, we wish to mention two other studies [19, 45] that explored aftereffect generalization across space. One study [19], using 66 dB sound level, found that aftereffect peaked at the adaptation location and clearly decreased moving away from there. The other study [45], using sound levels between 68.5 dB and 78.5 dB, found stronger spatial generalization: indeed, the aftereffect at the nontrained locations was as high as at the adaptation location. In the light of model hypothesis, such different results may be interpreted as the consequence of the different sound levels used in the two studies, given in particular the physiological evidence that sound level affects not only the spectral but also the spatial tuning function of the auditory neurons. Hence, differences in aftereffect generalization, both in the spectral and spatial domains, may have a common source.

## 5. Conclusions

In conclusion, we provide a biological plausible network able to reproduce a number of features of ventriloquism effect and aftereffect and to interpret, within a coherent framework, opposing experimental results as to aftereffect generalization across frequencies. In particular, the model suggests that auditory neurons in the cortex, spatially sensitive and frequency tuned—showing increase and expansion of their spatial and spectral tuning functions as auditory stimulus intensity increases—may be involved in ventriloquism effect and aftereffect. Biological neurons having these properties were observed in large proportion within the caudomedial field of auditory cortex. Caudomedial neurons have already been proposed to be involved in processing sound location information [23]; furthermore, neurons in this area have been shown to receive visual influences [41, 42]. According to the model, this auditory cortical area could be the functional site at which ventriloquism occurs and at which visual recalibration of auditory localization takes place. The inherent response properties of these neurons—inducing weaker and narrower activation at lower sound intensities and stronger and broader activation at higher sound intensities—may explain different extent of aftereffect generalization across frequencies, with no or little generalization at low sound intensities and wide generalization at high sound intensities.

The mechanisms hypothesized by the model to explain different extent of aftereffect generalization (i.e., differences in sound intensities) may be tested in vivo, by comparing model predictions with results of new experiments realized ad hoc. In particular, results reported in Figures 10 and 11, obtained with a wide range of combinations of the adaptation and testing intensities, represent testable predictions of the model that can be assessed experimentally using systematic combinations of appropriate physical sound levels. Experimental results in line with model predictions may support the validity of the model; possible discrepancies will be used to review and improve the model.

## Figures and Tables

**Table 1:** Basal values for network parameters.

Network dimensions	N_p_^v^ = 180	N_p_^a^ = 180	N_f_^a^ = 40			
External stimuli	E_0_^v^ = 15	σ_p_^v^ = 4	σ_p_^a^ = 30	σ_f_^a^ = 14		
Neurons response	*s* = 0.6	θ = 12	τ_y_ = 3 ms			
Visual basal synapses	L_ex0_^v^ = 2.4	L_in0_^v^ = 1.4	σ_ex,*p*_^v^ = 2	σ_in,*p*_^v^ = 24		
Auditory basal synapses	L_ex0_^a^ = 0.4	L_in0_^a^ = 0.21	σ_ex,*p*_^a^ = 1.45	σ_in,*p*_^a^ = 8	σ_ex,*f*_^a^ = 1.45	σ_in,*f*_^a^ = 5
Interlayer synapses	W_*av*_ = 8.5	W_*va*_ = 0.22				
Hebbian rules	θ_post_ = 0.5	α_ex0_ = 0.03	α_in0_ = 0.05	L_max⁡_^v^ = 2.4	L_max⁡_^a^ = 0.4	
